# Vibration Analysis of Fluid Conveying Carbon Nanotubes Based on Nonlocal Timoshenko Beam Theory by Spectral Element Method

**DOI:** 10.3390/nano9121780

**Published:** 2019-12-14

**Authors:** Xiaolei Yi, Baohui Li, Zhengzhong Wang

**Affiliations:** College of Water Resources and Architectural Engineering, Northwest A&F University, Yangling 712100, China; 18892119530@163.com (X.Y.); wangzz0910@163.com (Z.W.)

**Keywords:** fluid conveying SWCNT, nonlocal Timoshenko beam, spectral element method, dynamic response, natural frequency

## Abstract

In this work, we applied the spectral element method (SEM) to analyze the dynamic characteristics of fluid conveying single-walled carbon nanotubes (SWCNTs). First, the dynamic equations for fluid conveying SWCNTs were deduced based on the nonlocal Timoshenko beam theory. Then, the spectral element formulation was established for a free/forced vibration analysis of fluid conveying SWCNTs by introducing discrete Fourier transform. Furthermore, the proposed method was validated using several comparison examples. Finally, the natural frequencies and dynamic responses of a simply-supported fluid conveying SWCNTs were calculated by the SEM, considering different internal fluid velocities and small-scale parameters (SSPs). The effects of fluid velocity and SSPs on the dynamic characteristics of SWCNTs conveying fluid were revealed by the numerical results. Compared with other methods, the SEM shows high accuracy and efficiency.

## 1. Introduction

Carbon nanotubes (CNTs) have been extensively used in numerous areas due to their excellent mechanical properties [1,2]. A deep understanding of the mechanical behavior of CNTs leads to wider applications [3,4]. In general, a molecular dynamics simulation and experimental methods are usually adopted to predict the mechanical behavior of a CNT [5,6,7]. Since the experiments at the nanoscale level are difficult to conduct [8] and have high computational cost, the molecular dynamics simulation method is more often used to deal with small-size atomic systems. Hence, the continuum-based solid mechanics models or methods [8,9,10,11] bring vitality for predicting the mechanical properties of a large CNT. As an important mechanical property, the vibration characteristics of a CNT have become another hot topic for research in recent years. For example, Cai et al. [12,13] discussed the dynamic responses of a CNT-based nanobeam, which was considered a model of nanobalance. For slight vibrations, the deformation is linearly elastic and reversible. Therefore, elastic beam models can be adopted to analyze the dynamic properties of a CNT [14]. The conventional Euler-Bernoulli and Timoshenko beam theories, however, cannot be used directly to predict the vibration characters of CNTs because of the scale effect; this defect was overcome by the nonlocal elasticity theory proposed by Eringen et al. [15,16]. Based on nonlocal beam theory, the static bending, buckling, vibration, and wave properties of single-/double-walled CNTs have been studied systematically [17,18,19,20,21]. In recent years, the nonlocal mechanics of elastic nanobeams have undergone rapid progress, with a lot of newly-developed theories, e.g., strain-driven and stress-driven nonlocal integral elasticity theory [22,23,24], two-phase integral elasticity theory [25], nonlocal strain gradient elasticity theory [26,27,28], and modified nonlocal strain gradient elasticity theory [29,30].

When conveying fluid, CNTs have potential applications, such as medical conduits, sensors, and material processing [31]. In this problem, the interaction between the nanofluid and nanotube should be considered in a vibration analysis. Based on classical continuum mechanics theory, Stephanie et al. [32] investigated the effects of flow velocity on the damping, stability, and frequency shift of microscale resonators containing internal flow, and found that the microtubes were susceptible to instability by divergence and flutter. Starting from the dynamic theory of macropipe theory [33], Yoon et al. investigated the effects of internal flow on free vibrations and the instability of CNTs and the flow-induced flutter instability of cantilever CNTs [34,35]. They determined that surrounding the elastic medium can reduce the effect of the inner fluid on the natural frequencies, and even eliminate tube instability within a practical range of fluid velocity. The modified couple stress theory and nonlocal Euler–Bernoulli beam theory were successively employed by Wang [36,37,38] to study the vibration and instability of tubular micro- and nano- beams conveying fluid. He discussed the effect of nonlocal parameters on natural frequencies and the critical velocity of fluid conveying CNTs, and demonstrated that the effect on critical velocity can be neglected. Also, according to nonlocal elastic theory, Liang and Su [39] studied the vibration of fluid conveying CNTs with consideration of the internal fluid showing pulsating and viscous flow, and obtained stability regions in a frequency-amplitude plane. Considering the small-size effects and Knudsen number, the vertical nanotube conveying fluid was studied by Bahaadini et al. [40] based on the nonlocal strain gradient Timoshenko beam theory. A thermo-mechanical vibration instability analysis of the CNT conveying fluid was conducted by Afkhami and Farid [41] using a nonuniform cross-section nonlocal Timoshenko beam model. Besides a linear stability analysis, nonlinear vibration characteristics have also been focused on by researchers. Zhen and Fang [42] deduced the nonlinear equation of CNTs conveying fluid in terms of nonlocal Euler–Bernoulli beam theory and discussed the relationship between internal resonance and the axial external excitation force in detail. Considering the geometric nonlinearity and size-effect, Ghayesh et al. investigated the chaotic motions of nanofluid conveying nanotubes based on the nonlocal strain gradient theory and Beskok-Karniadakis theory [43]. Farjpour et al. developed the scale-dependent nonlinear model of viscoelastic nanotubes conveying fluid by means of nonlocal theory along with a strain gradient model [44]. The resonant response and the effects of several parameters on the nonlinear response are discussed in detail.

In this paper, based on the spectral element method (SEM), which was originally proposed by Doyle [45] for the analysis of macrostructure dynamics, we developed a method for the free/forced vibration analysis of a CNT conveying fluid. The dynamic equations of flow-induced vibrations of single-walled CNTs (SWCNTs) conveying fluid were deduced in terms of nonlocal Timoshenko beam theory, which suits a short, stubby structure. The nonlocal elasticity theory is of lower computational cost than other continuum-based solid mechanics models [8], so efficient numerical calculations are the goal of this paper. The spectral formulations for free/forced vibrations are established using discrete Fourier transform. Numerical examples were given to show the validity of the proposed SEM.

## 2. Dynamic Equations of SWCNT Conveying Fluid

**Figure 1:**
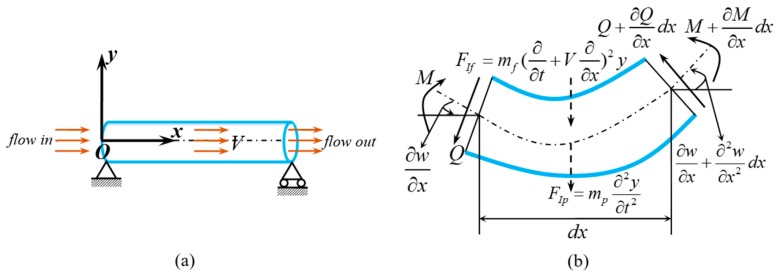
SWCNT conveying fluid. (**a**) Sketch of SWCNT conveying fluid. (**b**) Free body diagram.

The fluid conveying SWCNT with a free body diagram is shown in Figure 1. By assuming incompressible and non-viscous uniform flow, and considering the coupling effects (the centrifugal and Coriolis forces of fluid) between internal fluid and tube, the transverse vibration equation can be written as follows [46]:

(1) $$\frac{\partial Q}{\partial x}=m_{f}{\left(\frac{\partial }{\partial t}+V\frac{\partial }{\partial x}\right)}^{2}y+m_{p}\frac{\partial^{2}y}{\partial t^{2}}$$

where *V* is the velocity of the internal flow and *y* is the transverse deflection of the tube, i.e., the function of axial coordinate *x* and time *t*.

According to the nonlocal elastic theory, the dynamic equation of micro-Timoshenko beam takes the following form [19]:

(2) $$\left\{ \begin{array}{l} Q=k_{0}A_{p}G(\frac{\partial y}{\partial x}-\varphi )\\ M-{\left(e_{0}a\right)}^{2}\frac{\partial^{2}M}{\partial x^{2}}=EI\frac{\partial \varphi }{\partial x}\\ \frac{\partial M}{\partial x}+Q=J_{p}\frac{\partial^{2}\varphi }{\partial t^{2}} \end{array}\right.$$

where *E* is Young’s modulus, *G* is the shear modulus, *I* is the moment of inertia of the beam cross section about its neutral axis, *k*_0_ is the shear coefficient in Timoshenko beam theory, *μ* is the Poisson ratio, *A_p_* is the area of the beam cross section, *J_p_* is the mass moment of inertia, *m_p_* is the mass of tube per unit length, *m_f_* is the mass of fluid per unit length, and *e*_0_*a* is the scale coefficient to reflect a small-scale effect (Note: *e*_0_ is the material constant obtained by experiments or molecular dynamics, and *a* is the internal characteristic length, e.g., for CNT, the C–C bond length is usually chosen as the internal characteristic length [8,19]), *Q* is the shear force, *M* is the bending moment, and *φ* is the rotation angle of tube’s cross section caused by bending deformation.

According to Equations (1) and (2), the following partial differential equation can be obtained after eliminating *Q* and *M*:

(3) $$k_{0}A_{p}G(\frac{\partial^{2}y}{\partial x^{2}}-\frac{\partial \varphi }{\partial x})=(m_{p}+m_{f})\frac{\partial^{2}y}{\partial t^{2}}+2m_{f}V\frac{\partial^{2}y}{\partial x\partial t}+m_{f}V^{2}\frac{\partial^{2}y}{\partial t^{2}}$$

(4) $$EI\frac{\partial^{2}\varphi }{\partial x^{2}}+{\left(e_{0}a\right)}^{2}[J_{p}\frac{\partial^{4}\varphi }{\partial x^{2}\partial t^{2}}-k_{0}A_{p}G(\frac{\partial^{3}y}{\partial x^{3}}-\frac{\partial^{2}\varphi }{\partial x^{2}})]+k_{0}A_{p}G(\frac{\partial y}{\partial x}-\varphi )=J_{p}\frac{\partial^{2}\varphi }{\partial t^{2}}$$

By introducing the following non-dimensional parameters, the aforementioned equations will be relevantly simplified:

(5) $$\begin{array}{l} \eta =\frac{y}{L},\;\xi =\frac{x}{L},\;\alpha =\frac{e_{0}a}{L},\;u={\left(\frac{M}{EI}\right)}^{1/2}LV,\;\beta =\frac{m_{f}}{m_{f}+m_{p}},\;\\ \gamma =\frac{k_{0}A_{p}G}{EI}L^{2},\tau ={\left(\frac{EI}{m_{f}+m_{p}}\right)}^{1/2}\frac{t}{L^{2}},\;\sigma =\frac{J_{p}}{(m_{f}+m_{p})L^{2}},\;\\ \bar{M}=\frac{ML}{EI},\;\bar{Q}=\frac{QL^{2}}{EI},\;\omega ={\left(\frac{m_{f}+m_{p}}{EI}\right)}^{1/2}L^{2}\Omega \end{array}$$

where *L* is the length of the CNT.

Substituting the aforementioned terms in Equation (5) into Equations (3) and (4), one can obtain the dimensionless equations as follows:

(6) $$(\gamma -u^{2})\frac{\partial^{2}\eta }{\partial \xi^{2}}-\frac{\partial^{2}\eta }{\partial \tau^{2}}-2\sqrt{\beta }u\frac{\partial^{2}\eta }{\partial \xi \partial \tau }-\gamma \frac{\partial \varphi }{\partial \xi }=0$$

(7) $$\alpha^{2}\sigma \frac{\partial^{4}\varphi }{\partial \xi^{2}\partial \tau^{2}}-\gamma \alpha^{2}\frac{\partial^{3}\eta }{\partial \xi^{3}}+(1+\gamma \alpha^{2})\frac{\partial^{2}\varphi }{\partial \xi^{2}}+\gamma (\frac{\partial \eta }{\partial \xi }-\varphi )-\sigma \frac{\partial^{2}\varphi }{\partial \tau^{2}}=0$$

Taking the pairs of the discrete Fourier transform (Equation (8)) of *η* and *φ* back into Equations (6) and (7), the partial differential equations in the time domain can be transformed into ordinary differential equations in the frequency domain:

(8) $$\left\{ \begin{array}{l} \eta_{r}(\xi ,\tau_{r})=\sum_{n=0}^{N-1}{\hat{\eta }}_{n}(\xi ,\omega_{n})e^{i\omega_{n}\tau_{r}}\;(r=0,\;1,\;2,\;\cdots N-1)\\ {\hat{\eta }}_{n}(\xi ,\omega_{n})=\frac{1}{N}\sum_{r=0}^{N-1}\eta_{r}(\xi ,\tau_{r})e^{-i\omega_{n}\tau_{r}}\;(n=0,\;1,\;2,\;\cdots N-1) \end{array}\right.$$

where ${\hat{\eta }}_{n}$ is the discrete Fourier transform pair of *η_r_*, *ω* is the dimensionless form of frequency *Ω*, and the subscript in *ω_n_* presents the discrete dimensionless frequency. *N* denotes the number of samples adopted in the discrete Fourier transform, which is a kind of series expansion method [47]. In general, a more efficient fast Fourier transform (FFT) algorithm is used during the application. Accordingly, *N* needs to satisfy *N* = 2^n^ and n is a positive integer, *τ_r_* is the discrete dimensionless time, and *i* is an imaginary unit.

Substituting Equation (8) into Equations (6) and (7), one can get the ordinary differential equations with respect to ${\hat{\eta }}_{n}$ and ${\hat{\varphi }}_{n}$. Since these equations must be satisfied at every discrete frequency point, the subscript *n* can be omitted, and Equations (6) and (7) become

(9) $$(\gamma -u^{2})\frac{d^{2}\hat{\eta }}{d\xi^{2}}+\omega^{2}\hat{\eta }-2\sqrt{\beta }ui\omega \frac{d\hat{\eta }}{d\xi }-\gamma \frac{d\hat{\varphi }}{d\xi }=0$$

(10) $$-\alpha^{2}\sigma \omega^{2}\frac{d^{2}\hat{\varphi }}{d\xi^{2}}-\gamma \alpha^{2}\frac{d^{3}\hat{\eta }}{d\xi^{3}}+(1+\gamma \alpha^{2})\frac{d^{2}\hat{\varphi }}{d\xi^{2}}+\gamma (\frac{d\hat{\eta }}{\partial \xi }-\hat{\varphi })-\sigma \omega^{2}\hat{\varphi }=0$$

The solution of the ordinary differential Equations (9) and (10) with constant coefficients holds the following exponential form:

(11) $$\hat{\eta }(\xi )=Ce^{ik\xi },\;\hat{\varphi }(\xi )=De^{ik\xi }\;$$

where *k* is the dimensionless wave number, and *C*, *D* are undetermined coefficients.

Substituting Equation (11) into Equations (9) and (10), the following matrix equation can be obtained:

(12) $$\left[\begin{array}{cc} (u^{2}-\gamma )k^{2}+\omega^{2}+2\sqrt{\beta }ui\omega k & -\gamma ik\\ \gamma ik(\alpha^{2}k^{2}+1) & (\alpha^{2}\sigma \omega^{2}k-1-\alpha^{2}\gamma )k^{2}+\sigma \omega^{2}-\gamma \end{array}\right]\left[\begin{array}{c} C\\ D \end{array}\right]=\left[\begin{array}{c} 0\\ 0 \end{array}\right]$$

The dispersion relation of elastic wave propagation in SWCNT conveying fluid can be obtained using the nontrivial solution condition of Equation (12), i.e.,

(13) $$\begin{array}{l} \left[(u^{2}-\gamma )(\alpha^{2}\sigma \omega^{2}-1)-u^{2}\alpha^{2}\gamma ]k^{4}+2\sqrt{\beta }u\omega (\alpha^{2}\sigma \omega^{2}-\alpha^{2}\gamma -1)k^{3}+[u^{2}(\sigma \omega^{2}-\gamma \right)\\ -\gamma \sigma \omega^{2}+\omega^{2}(\alpha^{2}\sigma \omega^{2}-\alpha^{2}\gamma -1)]k^{2}+2\sqrt{\beta }u\omega (\sigma \omega^{2}-\gamma )k+\omega^{2}(\sigma \omega^{2}-\gamma )=0 \end{array}$$

The four roots of polynomial Equation (13) represent four dimensionless wave numbers. In general, half of them are real roots representing the propagating wave motion, and the other half are imaginary roots corresponding to evanescent wave motion. For one-dimensional wave propagation, one may use $k_{1},\;k_{2}$ denoting a left-traveling wave, and $k_{3},\;k_{4}$ a right-traveling wave. Due to the four wave numbers, Equation (10) can also be reformulated as follows:

(14) $$\hat{\eta }(\xi )=\sum_{j=1}^{4}C_{j}e^{ik_{j}\xi },\;\hat{\varphi }(\xi )=\sum_{j=1}^{4}D_{j}e^{ik_{j}\xi }\;$$

where $D_{j}=\lambda_{j}C_{j}$, and the coefficient $\lambda_{j}$ can be determined by Equation (12), i.e.,

(15) $$\lambda_{j}=\frac{i}{\gamma }[(\gamma -u^{2})k_{j}-2\sqrt{\beta }u\omega -\omega^{2}/k_{j}]$$

Similarly, both the dimensionless shear force and bending moment can be expressed as

(16) $$\hat{\bar{Q}}(\xi )=\sum_{j=1}^{4}\chi_{j}C_{j}e^{ik_{j}\xi },\;\hat{\bar{M}}(\xi )=\sum_{j=1}^{4}\zeta_{j}C_{j}e^{ik_{j}\xi }\;$$

with

(17) $$\left\{ \begin{array}{l} \chi_{j}=i(u^{2}k_{j}+2\sqrt{\beta }u\omega +\omega^{2}/k_{j})\\ \zeta_{j}=[(u^{2}-\gamma )k_{j}^{2}+2\sqrt{\beta }u\omega k_{j}+\omega^{2}]/(1+\alpha^{2}k_{j}^{2})/\gamma \end{array}\right.\;$$

Now, all the related parameters in the frequency-domain formulations are obtained. In the following section, the spectral formulation of a finite length SWCNT conveying fluid will be established.

## 3. Spectral Formulation of a SWCNT Conveying Fluid

### 3.1. The Spectral Formulations

**Figure 2:**
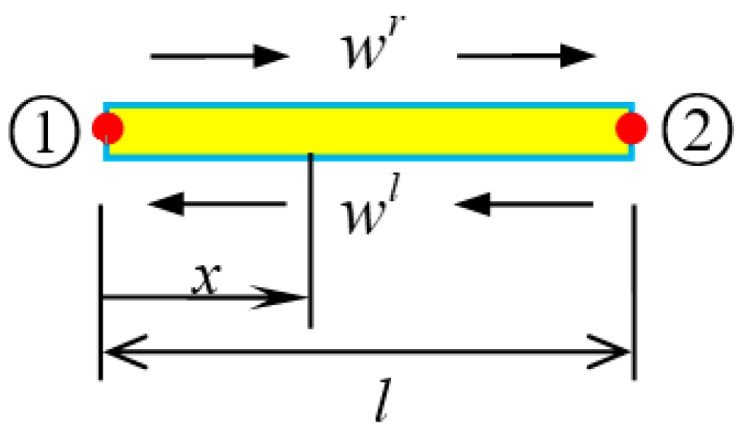
Sketch of wave motion in a SWCNT element.

The one-dimensional wave motion in SWCNT (Figure 2) includes left- and right-traveling waves $w^{l}$ and $w^{r}$. For a SWCNT element with length *l*, Equation (14) holds the following form if node 1 was chosen as origin [45]:

(18) $$\begin{array}{l} \hat{\eta }(\xi )=C_{1}e^{ik_{1}(\xi -1)}+C_{2}e^{ik_{2}(\xi -1)}+C_{3}e^{ik_{3}\xi }+C_{4}e^{ik_{4}\xi }\\ \;=\left[\begin{array}{cccc} e^{ik_{1}(\xi -1)} & e^{ik_{2}(\xi -1)} & e^{ik_{3}\xi } & e^{ik_{4}\xi } \end{array}\right]\;\left[\begin{array}{c} C_{1}\\ C_{2}\\ C_{3}\\ C_{4} \end{array}\right] \end{array}$$

**Figure 3:**
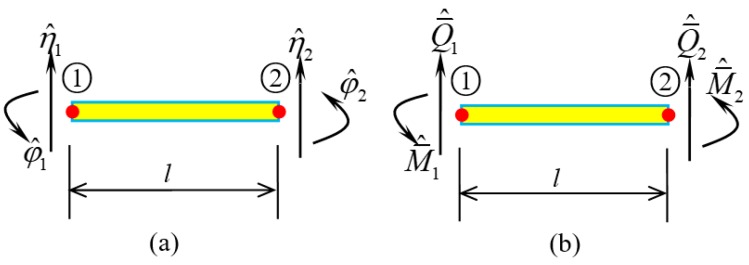
A SWCNT element. (**a**) Nodal displacements; (**b**) Nodal forces.

The nodal displacement (Figure 3a) of the element can be practically represented as.

(19) $$\left\{ \begin{array}{l} {\hat{\eta }}_{1}=\hat{\eta }(0),\;{\hat{\varphi }}_{1}=\hat{\varphi }(0)\\ {\hat{\eta }}_{2}=\hat{\eta }(1),\;{\hat{\varphi }}_{2}=\hat{\varphi }(1) \end{array}\right.$$

Putting a specific position parameter into Equations (18) and (19) can be formulated in a matrix form, i.e.,

(20) $$w^{\left(e\right)}={\left[\begin{array}{cccc} {\hat{\eta }}_{1} & {\hat{\varphi }}_{1} & {\hat{\eta }}_{2} & {\hat{\varphi }}_{2} \end{array}\right]}^{T}=A{\left[\begin{array}{cccc} C_{1} & C_{2} & C_{3} & C_{4} \end{array}\right]}^{T}$$

with

(21) $$A=\left[\begin{array}{cccc} e^{-ik_{1}} & e^{-ik_{2}} & 1 & 1\\ \lambda_{1}e^{-ik_{1}} & \lambda_{2}e^{-ik_{2}} & \lambda_{3} & \lambda_{4}\\ 1 & 1 & e^{ik_{3}} & e^{ik_{4}}\\ \lambda_{1} & \lambda_{2} & \lambda_{3}e^{ik_{3}} & \lambda_{4}e^{ik_{4}} \end{array}\right]$$

Substituting the coefficient column in Equation (20) into Equation (18) yields another expression of nodal displacements as follows:

(22) $$\hat{\eta }(\xi )=\left[\begin{array}{cccc} e^{ik_{1}(\xi -1)} & e^{ik_{2}(\xi -1)} & e^{ik_{3}\xi } & e^{ik_{4}\xi } \end{array}\right]\;A^{-1}w^{\left(e\right)}$$

In fact, Equation (22) presents the shape function of the spectral element. Similarly, the nodal force shown in Figure 3b reads

(23) $$\left\{ \begin{array}{l} {\hat{\bar{Q}}}_{1}=-\hat{\bar{Q}}(0),\;{\hat{\bar{Q}}}_{2}=\hat{\bar{Q}}(1)\\ {\hat{\bar{M}}}_{1}=-\hat{\bar{M}}(0),\;{\hat{\bar{M}}}_{2}=\hat{\bar{M}}(1) \end{array}\right.$$

In the same way, the nodal force can be written in a matrix form as follows:

(24) $$F^{\left(e\right)}={\left[\begin{array}{cccc} {\hat{\bar{Q}}}_{1} & {\hat{\bar{M}}}_{1} & {\hat{\bar{Q}}}_{2} & {\hat{\bar{M}}}_{2} \end{array}\right]}^{T}=B{\left[\begin{array}{cccc} C_{1} & C_{2} & C_{3} & C_{4} \end{array}\right]}^{T}$$

with

(25) $$B=\left[\begin{array}{cccc} -\chi_{1}e^{-ik_{1}} & -\chi_{2}e^{-ik_{2}} & -\chi_{3} & -\chi_{4}\\ -\zeta_{1}e^{-ik_{1}} & -\zeta_{2}e^{-ik_{2}} & -\zeta_{3} & -\zeta_{4}\\ \chi_{1} & \chi_{2} & \chi_{3}e^{ik_{3}} & \chi_{4}e^{ik_{4}}\\ \zeta_{1} & \zeta_{2} & \zeta_{3}e^{ik_{3}} & \zeta_{4}e^{ik_{4}} \end{array}\right]$$

Based on Equations (20) and (24), the relationship between the nodal force and nodal displacement can be obtained, i.e.,

(26) $$F^{\left(e\right)}=S^{\left(e\right)}(\omega )w^{\left(e\right)}$$

where matrix $S^{\left(e\right)}=BA^{-1}$ is called the spectral element matrix.

The natural frequency of the system can be obtained when Equation (26) has nontrivial solution, i.e., $\det\left|S^{\left(e\right)}\right|=0$. 

For a simply supported SWCNT, both ends are chosen as the element nodes, and the boundary conditions are as follows:

(27) $$\left\{ \begin{array}{l} {\hat{\eta }}_{1}=0,\;{\hat{\eta }}_{2}=0,\;{\hat{\varphi }}_{1}\neq 0,\;{\hat{\varphi }}_{2}\neq 0\\ {\hat{\bar{Q}}}_{1}\neq 0,\;{\hat{\bar{Q}}}_{2}\neq 0,\;{\hat{\bar{M}}}_{1}=0,\;{\hat{\bar{M}}}_{2}=0 \end{array}\right.$$

Substituting the aforementioned boundary conditions into Equation (26) and picking the related four elements in the matrix, one can get the following homogeneous equation:

(28) $$\left[\begin{array}{cc} S^{\left(e\right)}(2,\;2) & S^{\left(e\right)}(2,\;4)\\ S^{\left(e\right)}(4,\;2) & S^{\left(e\right)}(4,\;4) \end{array}\right]\;\left[\begin{array}{c} {\hat{\varphi }}_{1}\\ {\hat{\varphi }}_{2} \end{array}\right]=\left[\begin{array}{c} 0\\ 0 \end{array}\right]$$

In Equation (28), $S^{\left(e\right)}(i,\;j)$ denotes the element lying at the *i* th row and *j* th column of matrix $S^{\left(e\right)}$. Set $S_{s}^{\left(e\right)}=\left[\begin{array}{cc} S^{\left(e\right)}(2,\;2) & S^{\left(e\right)}(2,\;4)\\ S^{\left(e\right)}(4,\;2) & S^{\left(e\right)}(4,\;4) \end{array}\right]\;$, the natural frequencies can be calculated through a nontrivial solution condition of Equation (28), i.e.,

(29) $$\det\left|S_{s}^{\left(e\right)}\right|=0$$

### 3.2. Comparison Example

To validate the SEM, we calculated the first five natural frequency parameters $\sqrt{\omega }$ of SWCNT with two different boundary conditions and compared the results with those in reference [19]. The parameters of SWCNT are listed in Table 1, and the results of simply supported SWCNTs with different SSPs *α* are listed in Table 2.

There are several methods for computing the shear coefficient [48,49]. In this paper, the value of *k*_0_ can be obtained by $k_{0}=\frac{6(1+\mu ){\left(1+m\right)}^{2}}{(7+6\mu ){\left(1+m\right)}^{2}+(20+12\mu )m^{2}}$ for a Timoshenko beam with a hollow circle cross section [50,51].

It can be seen in Table 2 that the results in this paper are the same as those in reference [19]. Therefore, the SEM is available to predict the natural frequencies of a SWCNT in free vibration.

**Figure 4:**
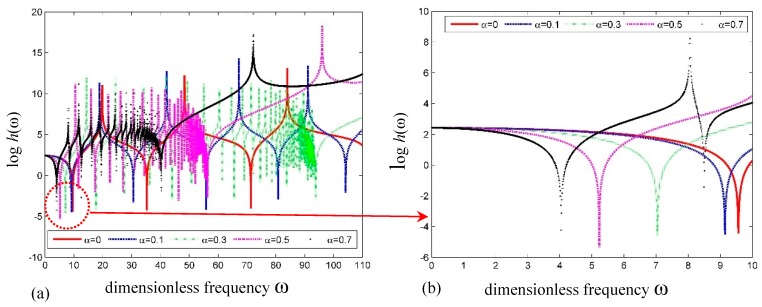
Curves of logh(ω) versus frequency for a simply supported SWCNT. (**a**) Global curve; (**b**) detailed curve.

Figure 4a,b shows the plots of frequency versus the log of determinant $h(\omega )=\det\left|S_{s}^{\left(e\right)}\right|$ for the simply supported SWCNT with different dimensionless small-scale parameters (SSPs), i.e., *α*. It can be seen that the natural frequencies decrease with increasing SSP. This means that the SWCNT becomes more flexible accordingly.

We also computed the first five natural frequency parameters $\sqrt{\omega }$ of a cantilevered SWCNT with four different SSPs *α*; the comparison results are shown in Table 3. The results by the SEM and in reference [19] are identical.

**Figure 5:**
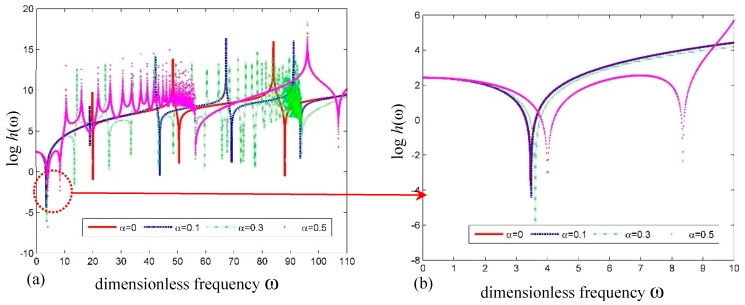
The plots of logh(ω) versus frequency for the cantilever SWCNT. (**a**) Global curve; (**b**) detailed curve.

The plots of frequency versus the log of determinant $h(\omega )=\det\left|S_{s}^{\left(e\right)}\right|$ for the cantilevered SWCNT with different dimensionless SSPs *α* are shown in Figure 5a,b. For the cantilevered SWCNT, the effect of *α* on the natural frequencies is slightly different from that on a simply supported SWCNT. It can be seen in Figure 5 that all the natural frequencies decrease with increasing *α*, except the first-order natural frequency, which actually increases.

### 3.3. Free Vibration of a SWCNT Conveying Fluid

In this example, we analyzed the free vibrations of a simply supported SWCNT conveying fluid with the same material parameters as those in Table 1. The density of the internal fluid was 1.0 g/cm^3^. For the purpose of investigating the effects of *α* and fluid velocity *u* on the natural frequencies, we calculated the first five natural frequencies of SWCNT conveying fluid in two cases, i.e., constant fluid velocity *u* = 0.4 with different SSPs *α* and constant *α* = 0.2 with different fluid velocities. The results of the former case are listed in Table 4, and those of the latter case are listed in Table 5.

**Figure 6:**
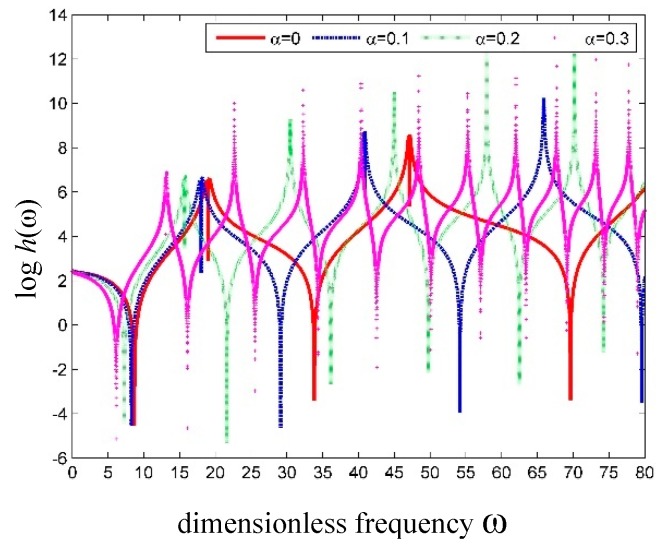
Curves of logh(ω) versus frequency for simply supported SWCNT conveying fluid with different SSPs.

As shown in Figure 6, the effect of *α* on the SWCNT conveying fluid is the same as that on the hollow SWCNT. All the natural frequencies decrease with increasing *α*.

**Figure 7:**
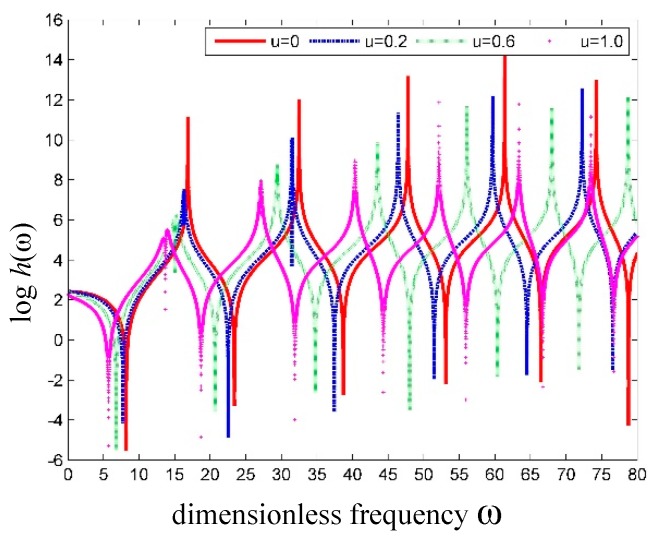
Cures of logh(ω) versus frequency for SWCNT conveying fluid with different velocities.

The effect of the internal fluid velocity on the natural frequencies of SWCNT conveying fluid, as shown in Figure 7, is the same as that of a macropipe conveying fluid. The flowing fluid weakens the tube effective stiffness and leads to a decrease in the natural frequencies. When the first natural frequency drops to zero, divergence (buckling) instability occurs; the corresponding velocity is named critical velocity.

**Figure 8:**
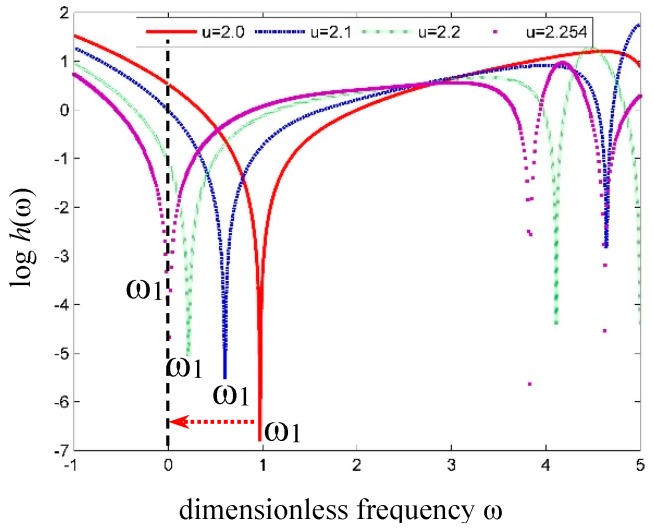
Curves of the fundamental frequency versus fluid velocity in a tube.

Moreover, the critical velocity of the simply supported fluid conveying SWCNTs was computed with the mass ratio *β* = 0.64, *α* = 0.3 to compare with that in reference [37]. The variation of the fundamental frequency with fluid velocity is shown in Figure 8. Herein, the critical velocity *u_c_* corresponding to zero fundamental frequency is *u_c_* = 2.254, which is slightly lower than that predicted by Wang [37] based on the nonlocal Euler-Bernoulli beam model with *u_c_* = 2.286. 

## 4. Dynamic Response of a Fluid Conveying SWCNTs

### 4.1. Spectral Formulations

**Figure 9:**
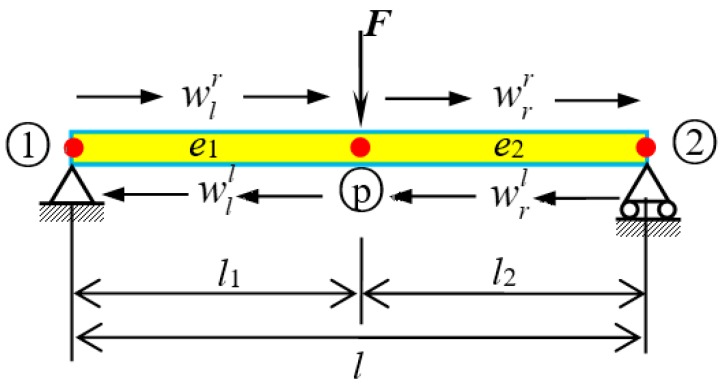
Simply supported SWCNT conveying fluid subjected to point load.

A simply supported SWCNT conveying fluid, as shown in Figure 9, was subjected to a point load in the middle part. *w* denotes the wave motion, while the subscripts *l* and *r* denote the left and right part of the load position or wave motion, respectively.

The SWCNT is divided into two elements when an external point load exists at the middle node p. Accordingly, the spectral matrix in Equation (26) is rewritten in the following global form:

(30) $$S^{\left(e\right)}=\left[\begin{array}{ccc} S_{11}^{\left(e_{1}\right)} & S_{12}^{\left(e_{1}\right)} & 0_{2\times 2}\\ S_{21}^{\left(e_{1}\right)} & S_{22}^{\left(e_{1}\right)}+S_{11}^{\left(e_{2}\right)} & S_{12}^{\left(e_{2}\right)}\\ 0_{2\times 2} & S_{21}^{\left(e_{2}\right)} & S_{22}^{\left(e_{2}\right)} \end{array}\right]$$

The nodal force and displacement column vectors are changed into the following form:

(31) $$\begin{array}{c} F^{\left(e\right)}={\left[\begin{array}{cccccc} {\hat{\bar{Q}}}_{1} & {\hat{\bar{M}}}_{1} & -\hat{\bar{F}} & 0 & {\hat{\bar{Q}}}_{2} & {\hat{\bar{M}}}_{2} \end{array}\right]}^{T}\\ w^{\left(e\right)}={\left[\begin{array}{cccccc} {\hat{\eta }}_{1} & {\hat{\varphi }}_{1} & {\hat{\eta }}_{p} & {\hat{\varphi }}_{p} & {\hat{\eta }}_{2} & {\hat{\varphi }}_{2} \end{array}\right]}^{T} \end{array}$$

For simplicity, the sequence of nodal force and displacement column vectors were rearranged as the following formulas [52]:

(32) $$\begin{array}{l} F_{R^{\prime }}^{\left(e\right)}={\left[\begin{array}{llllll} {\hat{\bar{Q}}}_{1} & {\hat{\bar{M}}}_{1} & {\hat{\bar{Q}}}_{2} & {\hat{\bar{M}}}_{2} & -\hat{\bar{F}} & 0 \end{array}\right]}^{T}={\left[\begin{array}{cc} F_{B} & F_{p} \end{array}\right]}^{T}\\ w_{R^{\prime }}^{\left(e\right)}={\left[\begin{array}{llllll} {\hat{\eta }}_{1} & {\hat{\varphi }}_{1} & {\hat{\eta }}_{2} & {\hat{\varphi }}_{2} & -{\hat{\eta }}_{p} & {\hat{\varphi }}_{p} \end{array}\right]}^{T}={\left[\begin{array}{cc} w_{B} & w_{p} \end{array}\right]}^{T} \end{array}$$

It is obvious that the relationship between the original and the current nodal force and displacement column vectors can be expressed in the following form by introducing the reordered matrix $R^{\prime }$ [52]:

(33) $$F^{\left(e\right)}=R^{\prime }F_{R^{\prime }}^{\left(e\right)},\;w^{\left(e\right)}=R^{\prime }w_{R^{\prime }}^{\left(e\right)}$$

where $R^{\prime }=\left[\begin{array}{cccccc} 1 & 0 & 0 & 0 & 0 & 0\\ 0 & 1 & 0 & 0 & 0 & 0\\ 0 & 0 & 0 & 0 & 1 & 0\\ 0 & 0 & 0 & 0 & 0 & 1\\ 0 & 0 & 1 & 0 & 0 & 0\\ 0 & 0 & 0 & 1 & 0 & 0 \end{array}\right]$, and ${R^{\prime }}^{T}R^{\prime }=I$.

The simply supported boundary conditions $\left\{ \begin{array}{l} {\hat{\eta }}_{1}=0,\;{\hat{\eta }}_{2}=0,\;{\hat{\varphi }}_{1}\neq 0,\;{\hat{\varphi }}_{2}\neq 0\\ {\hat{\bar{Q}}}_{1}\neq 0,\;{\hat{\bar{Q}}}_{2}\neq 0,\;{\hat{\bar{M}}}_{1}=0,\;{\hat{\bar{M}}}_{2}=0 \end{array}\right.$ can be modified in the following matric forms by introducing the boundary matrices $T_{b}$ and $T_{f}$ [52]:

(34) $$w_{B}=T_{b}{\left[\begin{array}{cc} {\hat{\varphi }}_{1} & {\hat{\varphi }}_{2} \end{array}\right]}^{T}=T_{b}{\bar{w}}_{B},\;F_{B}=T_{f}{\left[\begin{array}{cc} {\hat{\bar{Q}}}_{1} & {\hat{\bar{Q}}}_{2} \end{array}\right]}^{T}=T_{f}{\bar{F}}_{B}$$

with $T_{b}=\left[\begin{array}{cc} 0 & 0\\ 1 & 0\\ 0 & 0\\ 0 & 1 \end{array}\right],\;T_{f}=\left[\begin{array}{cc} 1 & 0\\ 0 & 0\\ 0 & 1\\ 0 & 0 \end{array}\right]$.

Now, the reordered nodal force and displacement vectors in Equation (22) can be rewritten as

(35) $$\begin{array}{l} F_{R^{\prime }}^{\left(e\right)}=\left[\begin{array}{cc} T_{f} & 0_{2\times 2}\\ 0_{2\times 2} & I_{2\times 2} \end{array}\right]\;\left[\begin{array}{c} {\bar{F}}_{B}\\ F_{p} \end{array}\right]={\bar{T}}_{f}F_{R^{\prime }}^{\left(e\right)}\\ w_{R^{\prime }}^{\left(e\right)}=\left[\begin{array}{cc} T_{b} & 0_{2\times 2}\\ 0_{2\times 2} & I_{2\times 2} \end{array}\right]\;\left[\begin{array}{c} {\bar{w}}_{B}\\ w_{p} \end{array}\right]={\bar{T}}_{b}{\bar{w}}_{R^{\prime }}^{\left(e\right)} \end{array}$$

Substituting Equations (33) and (35) into Equation (26) and multiplying both sides by ${R^{\prime }}^{T}$, the following results can be obtained:

(36) $${\bar{T}}_{f}{\bar{F}}_{R^{\prime }}^{\left(e\right)}={R^{\prime }}^{T}S^{\left(e\right)}R^{\prime }{\bar{T}}_{b}{\bar{w}}_{R^{\prime }}^{\left(e\right)}=S_{R^{\prime }}{\bar{T}}_{b}{\bar{w}}_{R^{\prime }}^{\left(e\right)}$$

With $S_{R^{\prime }}={R^{\prime }}^{T}S^{\left(e\right)}R^{\prime }$.

Multiplying both sides of Equation (36) by ${\bar{T}}_{b}^{T}$ and considering the result $T_{b}^{T}T_{f}=0_{2\times 2}$ [52], one can obtain the following result:

(37) $$\left[\begin{array}{c} 0_{2\times 1}\\ F_{p} \end{array}\right]={\bar{T}}_{b}^{T}S_{R^{\prime }}{\bar{T}}_{b}{\bar{w}}_{R^{\prime }}^{\left(e\right)}={\bar{S}}_{R^{\prime }}{\bar{w}}_{R^{\prime }}^{\left(e\right)}=\left[\begin{array}{cc} {\bar{S}}_{R^{\prime }11} & {\bar{S}}_{R^{\prime }12}\\ {\bar{S}}_{R^{\prime }21} & {\bar{S}}_{R^{\prime }22} \end{array}\right]{\bar{w}}_{R^{\prime }}^{\left(e\right)}$$

The unknown nodal displacement vector can be resolved from Equation (37) as follows [52]:

(38) $${\bar{w}}_{R^{\prime }}^{\left(e\right)}=\left[\begin{array}{c} {\bar{w}}_{B}\\ w_{p} \end{array}\right]=\left[\begin{array}{c} -{\bar{S}}_{R^{\prime }11}^{-1}{\bar{S}}_{R^{\prime }12}{\left({\bar{S}}_{R^{\prime }22}-{\bar{S}}_{R^{\prime }21}{\bar{S}}_{R^{\prime }11}^{-1}{\bar{S}}_{R^{\prime }12}\right)}^{-1}\\ {\left({\bar{S}}_{R^{\prime }22}-{\bar{S}}_{R^{\prime }21}{\bar{S}}_{R^{\prime }11}^{-1}{\bar{S}}_{R^{\prime }12}\right)}^{-1} \end{array}\right]F_{p}$$

Once the nodal displacement is obtained, the displacement at any location can be obtained according to the shape function expression of Equation (22):

(39) $$\hat{\eta }(\xi )=\left\{ \begin{array}{l} \left[\begin{array}{cccc} e^{ik_{1}(\xi -l_{1})} & e^{ik_{2}(\xi -l_{1})} & e^{ik_{3}\xi } & e^{ik_{4}\xi } \end{array}\right]\;A^{-1}w_{1}\;(0\leq \xi \leq l_{1})\\ \left[\begin{array}{cccc} e^{ik_{1}(\xi -l_{2})} & e^{ik_{2}(\xi -l_{2})} & e^{ik_{3}\xi } & e^{ik_{4}\xi } \end{array}\right]\;A^{-1}w_{2}\;(l_{1}\leq \xi \leq l_{2}) \end{array}\right.$$

with $w_{1}={\left[\begin{array}{cccc} 0 & {\hat{\varphi }}_{1} & {\hat{\eta }}_{p} & {\hat{\varphi }}_{p} \end{array}\right]}^{T},\;w_{2}={\left[\begin{array}{cccc} {\hat{\eta }}_{p} & {\hat{\varphi }}_{p} & 0 & {\hat{\varphi }}_{2} \end{array}\right]}^{T}$.

Equation (39) is the dynamic response of the SWCNT conveying fluid in the frequency domain, and that in the time domain can be quickly obtained through the inverse discrete Fourier transform of Equation (39).

### 4.2. Comparison Example

We also provided an example to demonstrate the validity of the SEM in dynamic response prediction. Up to now, no example of microbeams has been given; as such, the macro-Timoshenko beam was chosen here for comparison [53]. The beam with a rectangular cross-section is simply supported at both ends, and the point harmonic load is exerted at the center of the beam. The material parameters are listed in Table 6.

**Figure 10:**
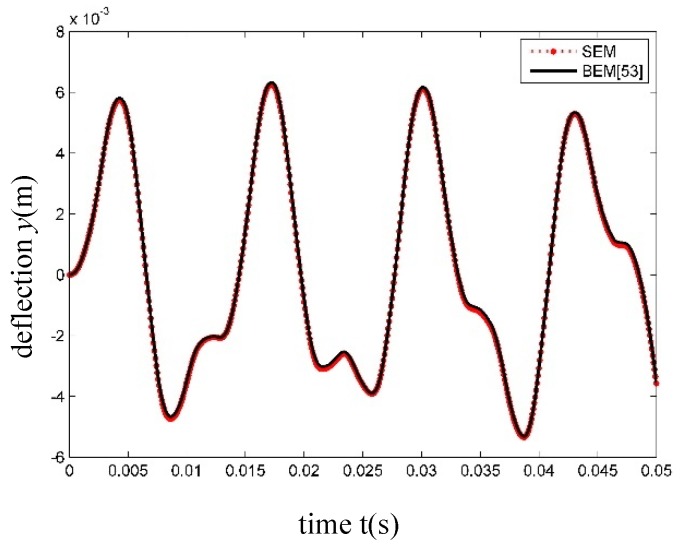
Time history of transverse displacement of Timoshenko beam at *x* = *L*/2 under harmonic load.

The steady harmonic load is $F(x,t)=10^{6}\delta (x-L/2)\sin(1000t)$, and the transverse displacement time history curves at *x* = *L*/2 are shown in Figure 10. Obviously, the results by the SEM are identical to those by the boundary element method (BEM) [53]. 

In reference [53], the time step was chosen as $\Delta t=5\times 10^{-6}$. However, the results with the same accuracy can be obtained using $\Delta t=1\times 10^{-4}$ in this work. Therefore, the SEM not only holds a high level of accuracy, but also takes low computational cost in dynamic analysis.

### 4.3. Example of SWCNT Conveying Fluid

We finally studied the dynamic response of a simply supported SWCNT conveying fluid under non-dimensional harmonic load $\bar{F}(\xi ,\tau )=\delta (\xi -1/2)\sin(\pi \tau )$ exerted on the midpoint of the tube. The same parameters of the same tube and internal fluid as those in Section 3.3 were used. Two cases with *u* = 0.4 and *α* = 0.2 are considered. 

**Figure 11:**
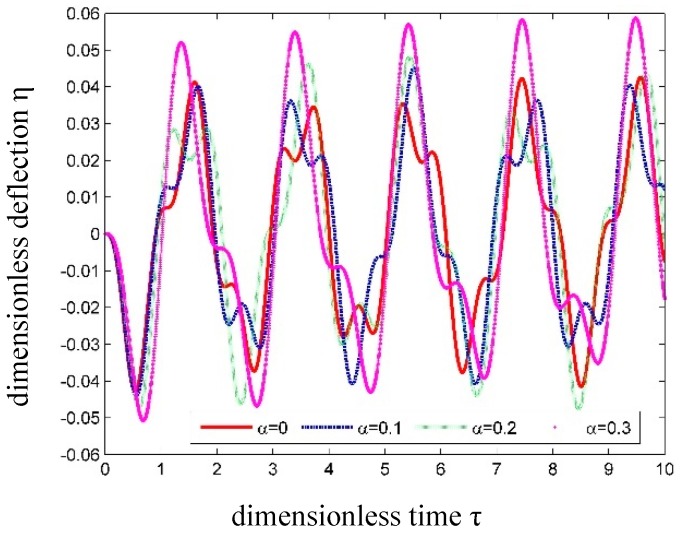
Time history curves of dimensionless transverse displacement at the center of SWCNT conveying fluid with different *α*.

When *u* = 0.4, the dimensionless transverse displacement at the center of the tube is drawn in Figure 11 with different *α* values.

As seen in Figure 11, the dynamic responses become larger as the SSP *α* increases. This effect of *α* on the SWCNT vibrations is the same as that in Section 3.3 for the free vibration situation. Such a phenomenon reflects the fact that the SWCNT will become more and more flexible if *α* increases. 

**Figure 12:**
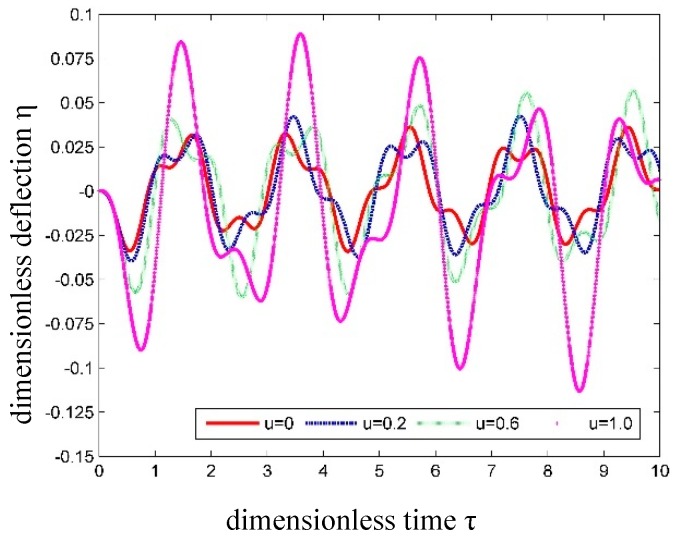
Time history curves of dimensionless transverse displacement at the center of SWCNT conveying fluid with different fluid velocities.

When *α* = 0.2, the time history of the dimensionless displacements is shown in Figure 12 for SWCNT conveying fluid with different fluid velocities.

In Figure 12, one can find that the internal fluid velocity has a significant influence on the dynamic response of SWCNT. This phenomenon can also be observed in the vibration of a macropipe conveying fluid. This influence, however, will not be as significant as that in a macropipe. The reason for this is that the mass of the fluid in SWCNT is lower, and the CNT has a relatively higher bending stiffness. So the instability of the SWCNT conveying fluid occurs only when the internal flow has an extremely high velocity.

## 5. Concluding Remarks

The SEM for the analysis of flow-induced vibration of a SWCNT conveying fluid was developed based on the nonlocal Timoshenko beam theory. The spectral formulations for free/forced vibration of SWCNT conveying fluid were established. Numerical examples were given to show the validity of the modified method. Some conclusions were drawn, as follows.

First, by calculating the first five natural frequencies for a SWCNT and the dynamic response for a macro-Timoshenko beam, the SEM is proved to be effective to predicts the dynamic characteristics of a SWCNT.

Second, the effect of *α* on the SWCNT conveying fluid is the same as that on the hollow SWCNT. All the natural frequencies decrease with increasing *α*.

Third, the effect of the internal fluid velocity on the natural frequencies of SWCNT conveying fluid is the same as that of a macropipe conveying fluid. The flowing fluid weakens the tube effective stiffness and leads to a decrease in the natural frequencies. When the flow velocity approaches the critical value, the first natural frequency drops to zero, and divergence (buckling) instability occurs.

Finally, compared with the BEM, the SEM can provide results with the same level of accuracy with a much larger time step, e.g., the time step in the present method is ~500 times higher than that in the BEM.

## Figures and Tables

**Table 1:** Parameters of SWCNT beam [19].

Young’s Modulus	Diameter	Length	CNT Density	Poisson Ration	Shear Coefficient
5.5 TPa	*d* = 0.678 nm	*L* = 10 *d*	2.3 g/cm^3^	*μ* = 0.19	*k*_0_ = 0.563

**Table 2:** The first five natural frequency parameters $\sqrt{\omega }$ of a simply supported SWCNT.

$\sqrt{\omega }$	*α* = 0		*α* = 0.1		*α* = 0.3		*α* = 0.5		*α* = 0.7	
	Present	[19]	Present	[19]	Present	[19]	Present	[19]	Present	[19]
$\sqrt{\omega_{1}}$	3.09	3.09	3.02	3.02	2.65	2.65	2.29	2.29	2.01	2.01
$\sqrt{\omega_{2}}$	5.94	5.94	5.53	5.53	4.21	4.21	3.40	3.40	2.92	2.92
$\sqrt{\omega_{3}}$	8.44	8.44	7.47	7.47	5.24	5.24	4.16	4.16	3.55	3.55
$\sqrt{\omega_{4}}$	10.63	10.63	8.99	8.99	6.02	6.02	4.74	4.74	4.03	4.03
$\sqrt{\omega_{4}}$	12.54	12.54	10.21	10.21	6.63	6.63	5.20	5.20	4.41	4.41

**Table 3:** The first five frequency parameters $\sqrt{\omega }$ of a cantilevered SWCNT.

$\sqrt{\omega }$	*α* = 0		*α* = 0.1		*α* = 0.3		*α* = 0.5	
	Present	[19]	Present	[19]	Present	[19]	Present	[19]
$\sqrt{\omega_{1}}$	1.86	1.86	1.87	1.87	1.90	1.90	2.00	2.00
$\sqrt{\omega_{2}}$	4.47	4.47	4.35	4.35	3.66	3.66	2.89	2.89
$\sqrt{\omega_{3}}$	7.11	7.11	6.61	6.61	5.08	5.08	-	-
$\sqrt{\omega_{4}}$	9.38	9.38	8.32	8.32	5.79	5.79	-	-
$\sqrt{\omega_{5}}$	11.38	11.38	9.67	9.67	6.58	6.58	-	-

**Table 4:** The first five natural frequencies of SWCNT conveying fluid when *u* = 0.4.

ω	*α* = 0	*α* = 0.1	*α* = 0.2	*α* = 0.3
$\omega_{1}$	8.69	8.26	7.27	6.14
$\omega_{2}$	33.82	29.10	21.61	16.09
$\omega_{3}$	69.61	54.16	36.12	25.53
$\omega_{4}$	111.14	79.56	49.76	34.33
$\omega_{5}$	155.37	104.07	62.67	42.53

**Table 5:** The first five natural frequencies of the SWCNT conveying fluid with *α* = 0.2.

ω	*u* = 0	*u* = 0.2	*u* = 0.6	*u* = 1.0
$\omega_{1}$	8.18	7.74	6.77	5.70
$\omega_{2}$	23.38	22.51	20.67	18.69
$\omega_{3}$	38.69	37.43	34.76	31.89
$\omega_{4}$	53.08	51.45	48.01	44.32
$\omega_{5}$	66.45	64.50	60.36	55.92

**Table 6:** Parameters of a Timoshenko beam [53].

Young’s Modulus	Density	Length	Section Size	Poisson Ratio	Shear Coefficient
50 GPa	2.5 g/cm^3^	4 m	*b* = 0.2 m *h* = 0.6 m	*μ* = 0.2	*k*_0_ = 5/6
